# Supplementary material for: Gustatory thalamic neurons mediate aversive behaviors
Source: Nat Commun. 2025 Sep 26;16:8517. doi: 10.1038/s41467-025-63464-5 (PMC12475432; doi:10.1038/s41467-025-63464-5)
Supplement: Supplementary file 1 — Supplementary Information [file 41467_2025_63464_MOESM1_ESM.pdf]

Supplementary information

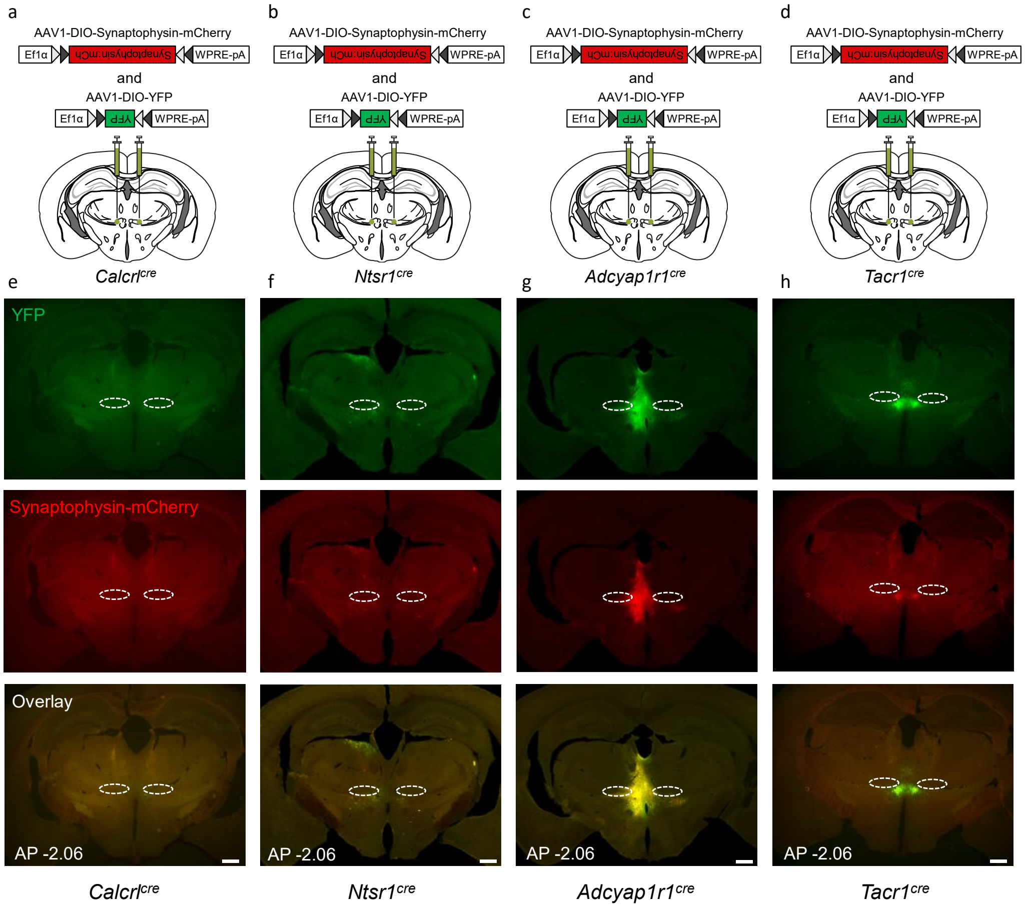

Supplementary Fig. 1 YFP and Synaptophysin expression in VPMpc of *Calcrl*^{Cre}, *Ntsr1*^{Cre}, *Adcyap1r1*^{Cre} or *Tacr1*^{Cre} mice

a-d, Scheme showing bilateral injection of AAV1-DIO-mYFP and AAV1-DIO-Synaptophysin:mCherry into the VPMpc of *Calcrl*^{cre} (**a**), *Ntsr1l*^{cre} (**b**), *Adcyap1r1*^{cre} (**c**), and *Tacr1l*^{cre} (**d**) mice. **e**, YFP (top), Synaptophysin:mCherry (middle) expression in the VPMpc of *Calcrl*^{cre} mice. **f**, YFP (top), Synaptophysin:mCherry (middle) expression in the VPMpc of *Ntsr1l*^{cre} mice. **g**, YFP (top), Synaptophysin:mCherry (middle) expression in the VPMpc of *Adcyap1r1*^{cre} mice. **h**, YFP (top), Synaptophysin:mCherry (middle) expression in the VPMpc of *Tacr1l*^{cre} mice. Scale bar 500 µm. AP: anterior-posterior bregma level, dotted circle is the VPMpc.

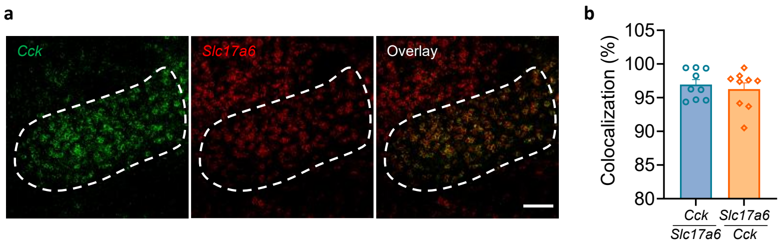

Supplementary Fig. 2 Colocalization of *Cck* and *Slc17a6* in VPMpc

a, Sample images showing RNAscope of *Cck* and *Slc17a6* in VPMpc of WT mice, scale bar 100 µm. **b**, Summary data showing the colocalization of *Cck* (n=9) and *Slc17a6* (n=9).

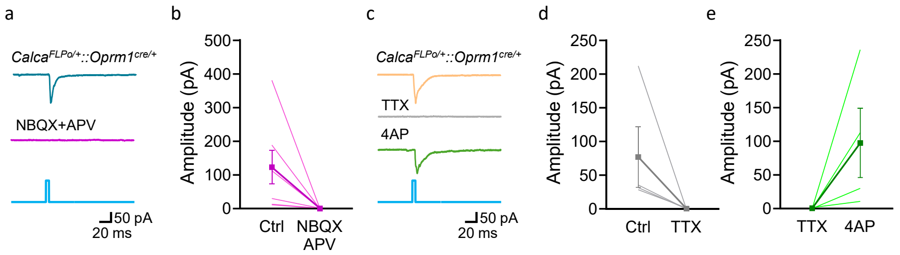

Supplementary Fig. 3 CGRP^{PBN} neurons send direct excitatory input to OPRM1^{VPMpc} neurons

a, Sample traces and **b**, summary figure of EPSCs in the VPMpc neurons of *Calca^{FLPo}::Oprm1^{Cre}* evoked by 470-nm blue light illumination and blocked by NBQX and APV (n = 7 per group). **c**, Sample traces and summary figures of EPSCs in the VPMpc neurons of *Calca^{FLPo}::Oprm1^{Cre}* evoked by 470-nm blue light illumination, **d**, blocked by TTX and **e**, reinstated by 4AP (n = 4 per group). Data are represented as mean ± SEM.

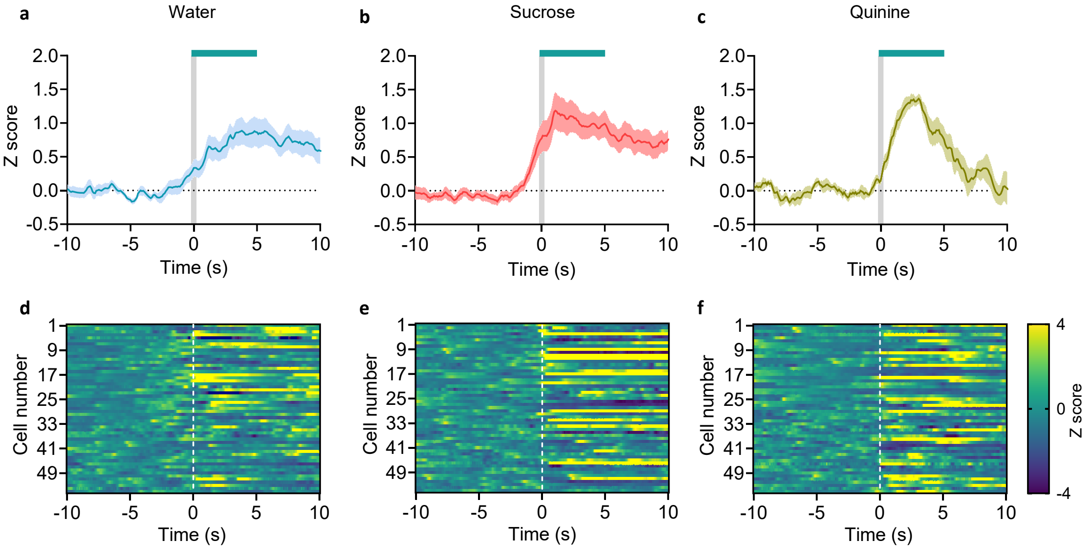

Supplementary Fig. 4 VPMpc neurons respond to taste stimuli

a-c, Average traces of all recorded CCK^{VPMpc} neuron calcium fluorescence in response to 5-s access of **a**) water, **b**) 5% sucrose and **c**) 0.1 mM quinine. **d-f**, Heat maps of individual neuronal responses in response to **d**) water, **e**) 5% sucrose and **f**) 0.1 mM quinine. Vertical dashed lines: onset of each stimulus. Data are represented as mean ± SEM.

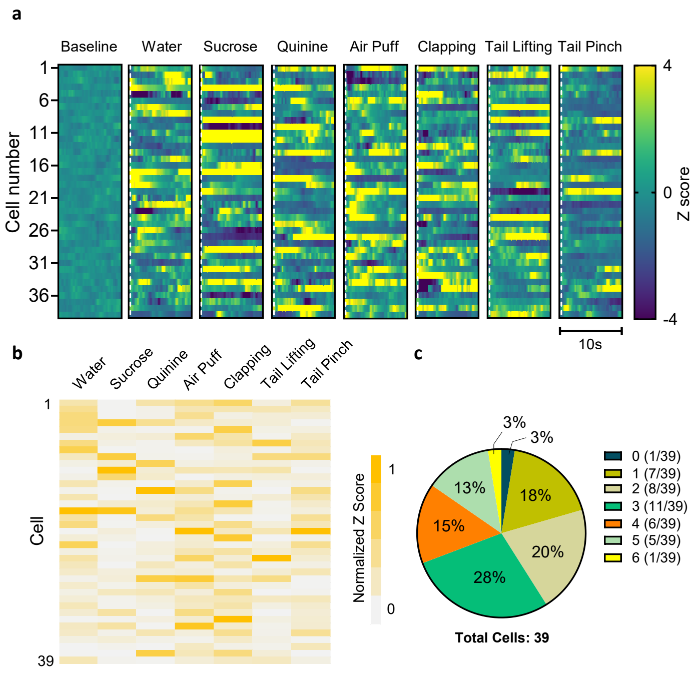

Supplementary Fig. 5 Individual neuronal responses across taste and sensory stimuli

a, Heat maps of all 39 CCK^{VPMpc} neurons showing individual neuronal responses of each cell to seven different stimuli, arranged in order of 5-s access of water, 5-s access of 5% sucrose, 5-s access of 0.1mM quinine, air puff, clapping, tail lifting and tail pinch. Vertical dashed lines: onset of each stimulus. **b**, Absolute value of the average response across seven different stimuli in the first 10 s after the stimulation onset, arranged the same order as in **a** and normalized to the maximum response of individual stimulus. **c**, Percent of cells that respond to each of the seven stimuli.

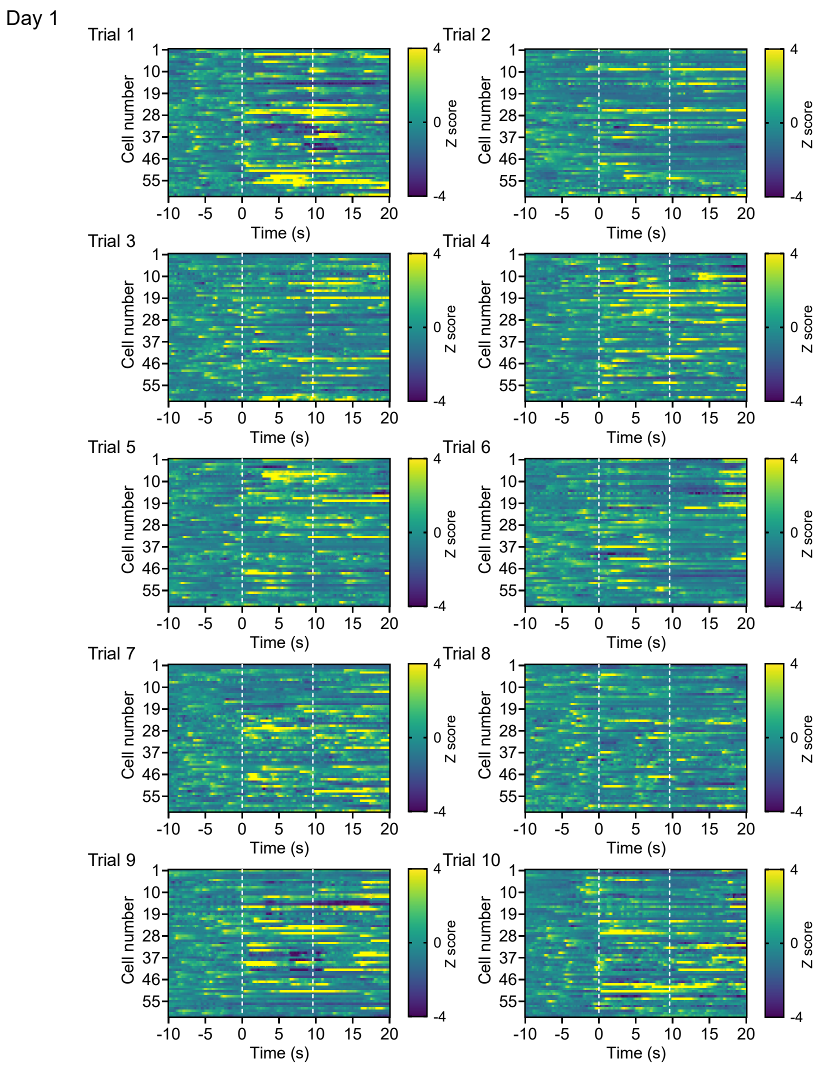

Supplementary Fig. 6 Heat maps of calcium fluorescence activity during 10 trials of 30 s calcium recordings in Day 1

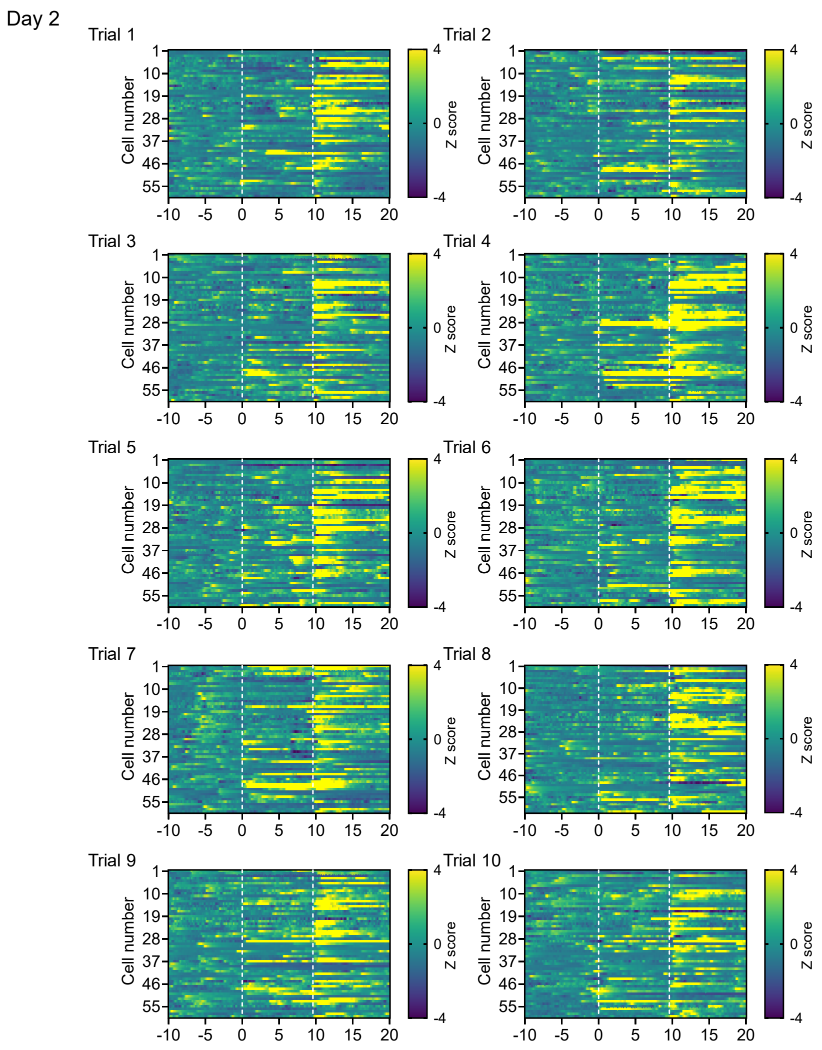

Supplementary Fig. 7 Heat maps of calcium fluorescence activity during 10 trials of 30 s calcium recordings in Day 2

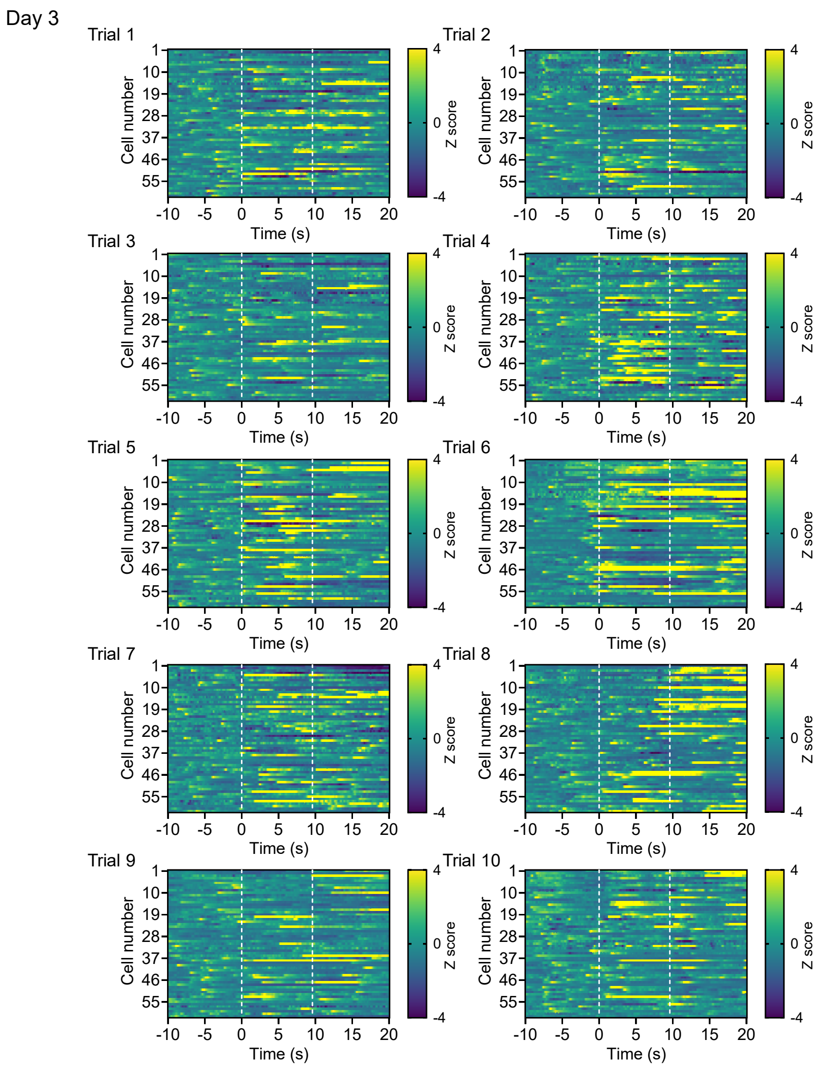

Supplementary Fig. 8 Heat maps of calcium fluorescence activity during 10 trials of 30 s calcium recordings in Day 3

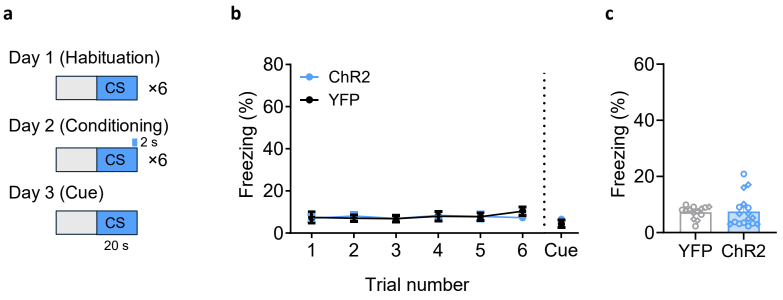

Supplementary Fig. 9 Photo-stimulating VPMpc cannot elicit fear learning

a, Experimental paradigm for the 3-day, cue-dependent optogenetic conditioning. 2-s unilateral photo-stimulation (30 Hz, 10 ms, 15 mW) was used to substitute for the foot shock as the unconditional stimulus. **b**, Summary data showing photo-stimulating of OPRM1^{VPMpc} neurons was unable to serve as an unconditional stimulus and did not elicit freezing response in either YFP- (n=14) or ChR2-expressing mice (n=17). **c**, Summary data showing no freezing response of ChR2-expressing mice in context test 24 h after conditioning (YFP: n=14, ChR2: n=17). Circled individual data points represent *Cck*^{Cre} mice, diamonds represent *Oprm1*^{Cre} mice in the summarized bar graphs.

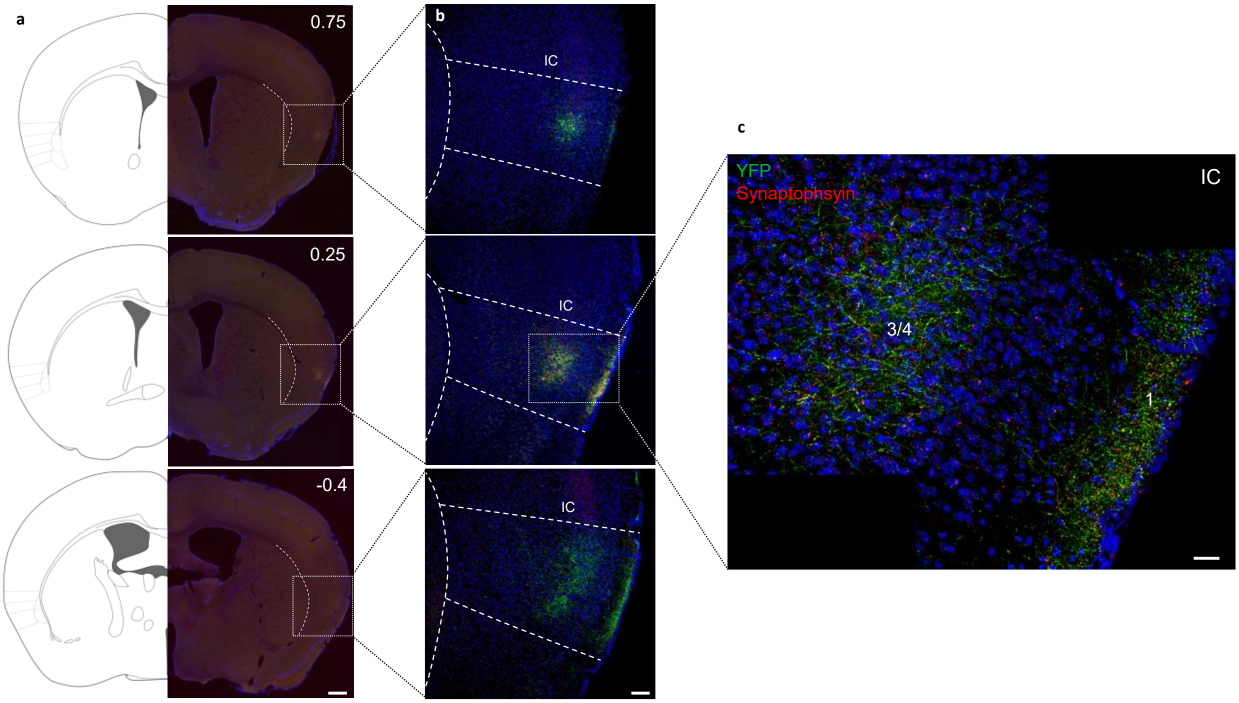

Supplementary Fig. 10 CCK^{VPMpc} neurons send axonal projection to IC

a, Coronal map (left) and slice Images (right) showing VPMpc axon terminals in different bregma levels containing the IC, scale bar 500 µm. **b**, Higher magnification images of the white box in (**a**) showing VPMpc axon terminals in different bregma levels of IC, scale bar 100 µm. **c**, Zoom-in images of the white box in (**b**) showing VPMpc axon terminals expressed in IC (bregma 0.25), scale bar 25 µm.

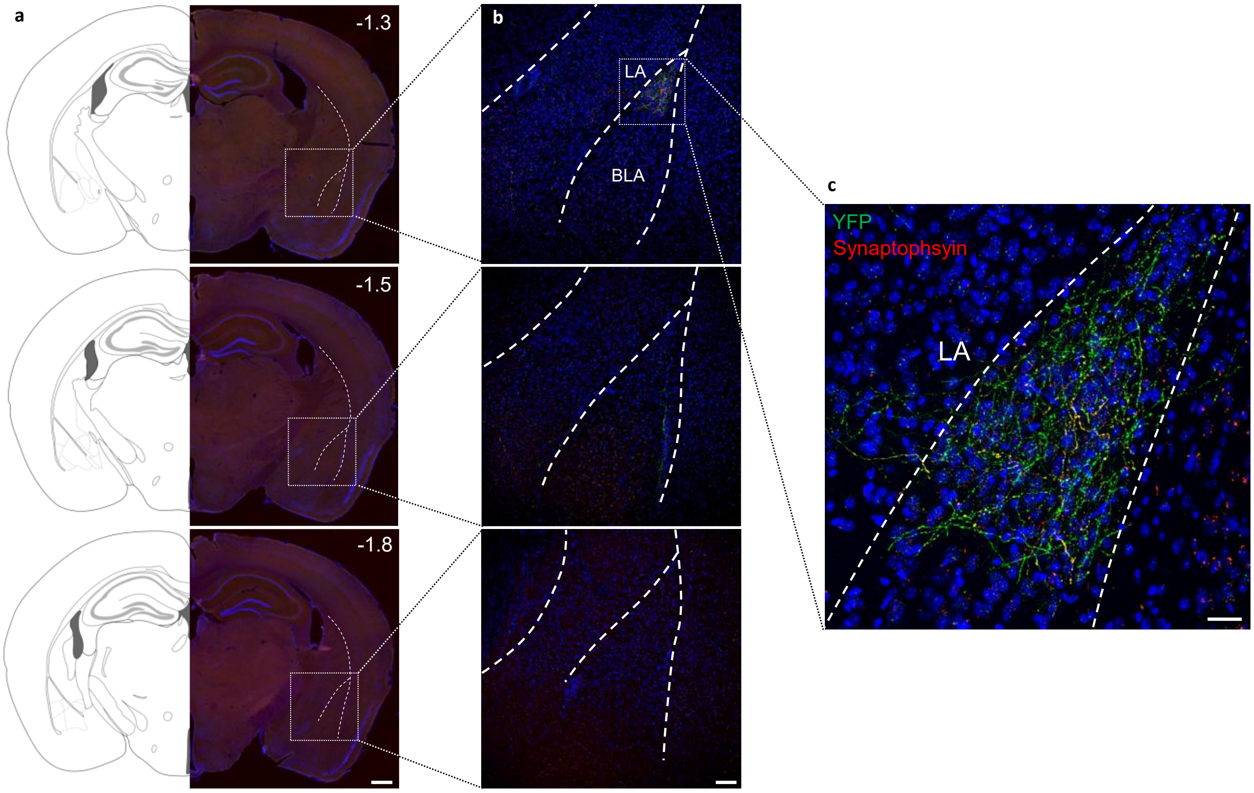

Supplementary Fig. 11 CCK^{VPMpc} neurons send axonal projection to rostral LA

a, Coronal map (left) and slice Images (right) showing VPMpc axon terminals in different bregma levels containing the LA, scale bar 500 µm. **b**, Higher magnification images of the white dash box in (**a**) showing VPMpc axon terminals in different bregma levels of LA, scale bar 100 µm. **c**, Zoom-in images of the white dash box in (**b**) showing VPMpc axon terminals only expressed in rostral LA (bregma -1.3), scale bar 25 µm.

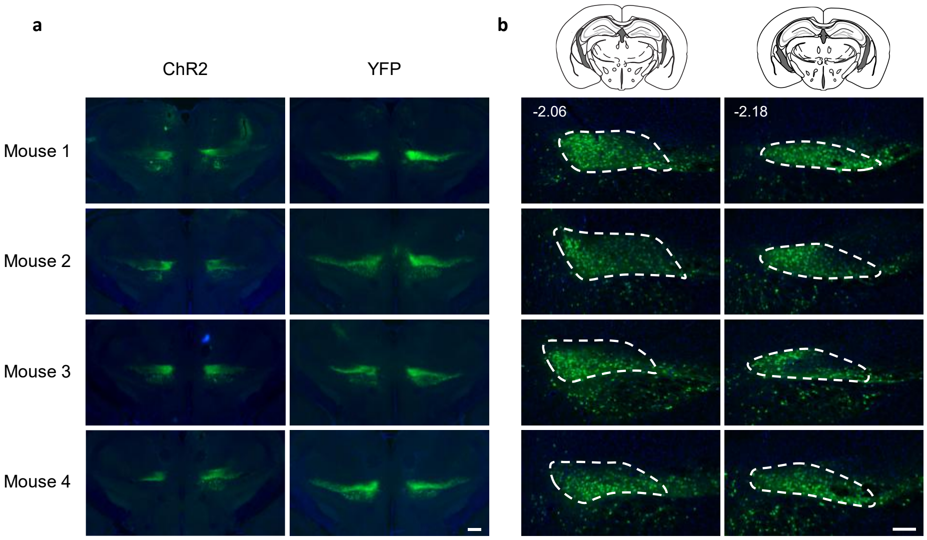

Supplementary Fig. 12 Viral expression in VPMpc

a, Sample images showing AAV1-DIO-ChR2:YFP and AAV1-DIO-YFP expression in the VPMpc of *Cck*^{Cre} mice, scale bar 400 µm **b**, Sample images showing higher magnification of YFP expression in two different bregma levels of the VPMpc in *Cck*^{Cre} mice, scale bar 150 µm.

Supplementary Table. 1 Statistical analysis summary sheets

Figure	Group (n number)	Shapiro-Wilk test (P>0.05 passed normality test)	Statistical test	P value
Fig. 1e	Cck/Oprm1 (n=21)	<0.0001	Mann Whitney test (Two-tailed) U=185	*P*=0.1917
	Oprm1/Cck (n=21)	<0.0001		
Fig. 1g	Cck/Oprm1 (n=21)	0.4977	Unpaired t test (Two-tailed) t=0.0407, df=39.98	*P*=0.9677
	Oprm1/Cck (n=21)	0.435		
Fig. 1h	mCherry (n=9)	0.0006	Mann Whitney test (Two-tailed) U=0	*P*<0.0001
	hM3Dq (n=12)	0.5789		
Fig. 1m	Cck^{cre} (n=13)	0.0212	Mann Whitney test (Two-tailed) U=259	*P*=0.7013
	Oprm1^{cre} (n=43)	<0.0001		
Fig. 4b	YFP (n=10)	0.0001	Mann Whitney test (Two-tailed) U=3	*P*<0.0001
	TetTox (n=9)	0.1648		
Fig. 4c	YFP (n=11)	0.5506	Unpaired t test (Two-tailed) t=2.999, df=19.94	*P*=0.0071
	TetTox (n=14)	0.1226		
Fig. 4d	YFP (n=11)	0.6386	Mann Whitney test (Two-tailed) U=36.5	*P*=0.0254
	TetTox (n=14)	0.0051		
Fig. 4f	Shock training			
	YFP (n=13)	0.9745	Two-way RM ANOVA with Holm-Šídák's multiple comparisons test F (1, 24) = 66.93	*P*<0.0001
	TetTox (n=13)	0.8061		
	Holm-Šídák's multiple comparisons test		Trial 1 t=1.653, df=19.71	*P*=0.1142
			Trial 2 t=3.279 df=15.24	*P*=0.0099
			Trial 3 t=4.596 df=15.24	*P*=0.0010
			Trial 4 t=6.893 df=18.33	*P*<0.0001
			Trial 5 t=5.733 df=20.83	*P*<0.0001
			Trial 6 t=4.832 df=22.31	*P*=0.0003
	Cue test			
	YFP (n=13)	0.7198	Unpaired t test (Two-tailed) t=4.741, df=21.42	*P*=0.0001
	TetTox (n=13)	0.0515		
Fig. 4g	Context test			
	YFP (n=13)	0.1125	Mann Whitney test (Two-tailed) U=12	*P*<0.0001
	TetTox (n=13)	0.0143		
Fig. 4i	YFP (n=12)	0.2602	Two-way RM ANOVA F (1, 24) = 0.0122	*P*=0.9128
	TetTox (n-=14)	0.8862		

Fig. 4j	Baseline			
	YFP (n=12)	0.7798	Unpaired t test (Two-tailed) t=0.8751, df=21.00	*P*=0.3914
	TetTox (n=11)	0.44		
	Test			
	YFP (n=12)	0.7078	Unpaired t test (Two-tailed) t=2.378, df=14.70	*P*=0.0314
	TetTox (n=11)	0.5401		
Fig. 4m	YFP			
	Saline (n=4)	0.0521	Paired t test (Two-tailed) t=3.396, df=3	*P*=0.0426
	U50 (n=4)	0.0521		
	TetTox			
	Saline (n=6)	0.6669	Paired t test (Two-tailed) t=0.3047, df=5	*P*=0.7729
	U50 (n=6)	0.6669		
Fig. 5c	Ctrl (n=5)	0.3714	Unpaired t test (Two-tailed) t=3.011, df=7	*P*=0.0196
	hM3Dq (n=4)	0.5108		
Fig. 5d	Ctrl (n=10)	0.1719	Mann Whitney test (Two-tailed) U=12	*P*=0.0025
	hM3Dq (n=10)	0.0176		
Fig. 5e	Ctrl (n=9)	0.5654	Unpaired t test (Two-tailed) t=2.812, df=12.54	*P*=0.0151
	hM3Dq (n=7)	0.0673		
Fig. 5f	Ctrl (n=9)	0.4213	Unpaired t test (Two-tailed) t=3.004, df=13.49	*P*=0.0098
	hM3Dq (n=7)	0.2308		
Fig. 5i	YFP (n=10)	0.552	Two-way RM ANOVA with Holm-Šídák's multiple comparisons test F (1, 18) = 11.45	*P*=0.0033
	ChR2 (n=10)	0.0443		
	Holm-Šídák's multiple comparisons test		Time Bin 1 t=0.1574, df=17.16	*P*=0.999
			Time Bin 2 t=1.28, df=17.86	*P*=0.8587
			Time Bin 3 t=4.355, df=17.78	*P*=0.0039
			Time Bin 4 t=0.2288, df=17.34	*P*=0.999
			Time Bin 5 t=1.014, df=17.96	*P*=0.9354
			Time Bin 6 t=5.184. df=17.25	*P*=0.0008
			Time Bin 7 t=0.5143, df=16.2	*P*=0.9967
			Time Bin 8 t=0.024, df=17.81	*P*=0.9996
			Time Bin 9 t=4.015, df=16.61	*P*=0.0084
			Time Bin 10 t=0.4869, df=17.99	*P*=0.9967
			Time Bin 11 t=0.0225, df=15.45	*P*=0.9996
Fig. 5j	YFP (n=6)	0.3235		*P*=0.0032

	ChR2 (n=8)	0.2971	Two-way RM ANOVA with Holm-Šídák's multiple comparisons test F (1, 12) = 13.50	
	Holm-Šídák's multiple comparisons test		Day (-1) t=0.91, df=10.05	*P*=0.6207
			Day (0) t=4.791, df=5.953	*P*=0.0123
			Day (1) t=5.593, df=6.063	*P*=0.0067
			Day (2) t=3.202, df=6.656	*P*=0.0475
			Day (3) t=0.2926, df=11.98	*P*=0.7749
Fig. 5m	YFP			
	Blank (n=14)	0.3971	Paired t test (Two-tailed) t=0.082, df=13	*P*=0.9359
	Spot (n=14)	0.3971		
	ChR2			
	Blank (n=16)	0.3509	Paired t test (Two-tailed) t=0.3091, df=15	*P*=0.7615
	Spot (n=16)	0.3509		
Fig. 5n	YFP			
	On (n=14)	0.7492	Paired t test (Two-tailed) t=0.5306, df=13	*P*=0.6047
	Off (n=14)	0.8308		
	ChR2			
	On (n=16)	0.3765	Paired t test (Two-tailed) t=15.9, df=13	*P*<0.0001
	Off (n=16)	0.7568		
Fig. 6h	IC (n=17)	0.0742	Unpaired t test (Two-tailed) t=1.574, df=33.83	*P*=0.1248
	LA (n=23)	0.2652		
Fig. 7e	YFP (n=4)	0.587	Two-way RM ANOVA F (1, 8) = 12.91	*P*=0.0071
	ChR2 (n=6)	0.0498		
Fig. 7f	YFP (n=4)	0.6502	Two-way RM ANOVA F (1, 8) = 44.98	*P*=0.0002
	ChR2 (n=6)	0.0058		
Fig. 7h	YFP (n=4)			
	Blank	0.0212	Wilcoxon matched-pairs signed rank test (Two-tailed) W=2	*P*=0.875
	Spot	0.0212		
	ChR2 (n=6)			
	Blank	0.3971	Paired t test (Two-tailed) t=0.0478, df=5	*P*=0.9637
	Spot	0.3971		
Fig. 7i	YFP (n=4)			
	On	0.8808	Paired t test (Two-tailed) t=0.2405, df=3	*P*=0.8254
	Off	0.8808		

	ChR2 (n=6)			
	On	0.1378	Paired t test (Two-tailed)<br>t=4.712, df=5	*P*=0.0053
	Off	0.1378		
Fig. 7j	YFP (n=4)			
	On	0.225	Paired t test (Two-tailed)<br>t=0.2762, df=3	*P*=0.8003
	Off	0.225		
	ChR2 (n=6)			
	On	0.8414	Paired t test (Two-tailed)<br>t=5.861, df=5	*P*=0.002
	Off	0.8414		
Fig. 7l	YFP (n=4)	0.8369	Two-way RM ANOVA<br>F (1, 7) = 13.24	*P*=0.0083
	ChR2 (n=5)	0.2031		
Fig. 7m	YFP (n=4)	0.0591	Two-way RM ANOVA<br>F (1, 7) = 29.46	*P*=0.0010
	ChR2 (n=5)	0.0089		
Fig. 7o	YFP (n=4)			
	Blank	0.1855	Paired t test (Two-tailed)<br>t=1.255, df=3	*P*=0.2984
	Spot	0.1855		
	ChR2 (n=4)			
	Blank	0.9887	Paired t test (Two-tailed)<br>t=1.358, df=3	*P*=0.2675
	Spot	0.9887		
Fig. 7p	YFP (n=4)			
	On	0.4813	Paired t test (Two-tailed)<br>t=0.253, df=3	*P*=0.8166
	Off	0.4813		
	ChR2 (n=4)			
	On	0.0618	Paired t test (Two-tailed)<br>t=0.0998, df=3	*P*=0.9268
	Off	0.0618		
Fig. 7q	YFP (n=4)			
	On	0.9287	Paired t test (Two-tailed)<br>t=1.711, df=3	*P*=0.1855
	Off	0.9287		
	ChR2 (n=4)			
	On	0.2996	Paired t test (Two-tailed)<br>t=1.055, df=3	*P*=0.3689
	Off	0.2996		
Supplementary Fig.2b	Cck (n=9)	0.0664	Unpaired t test (Two-tailed)<br>t=0.5866, df=14.99	*P*=0.5662
	Slc17a6 (n=9)	0.09		
Supplementary Fig.3b	Ctrl (n=7)	0.1094	Paired t test (Two-tailed)<br>t=2.448, df=6	*P*=0.0499
	NBQX/APV (n=7)	0.5756		

Supplementary Fig.3d	Ctrl (n=4)	0.0036	Wilcoxon matched-pairs signed rank test (Two-tailed) W=-10	*P*=0.125
	TTX (n=4)	0.009		
Supplementary Fig.3e	TTX (n=4)	0.009	Wilcoxon matched-pairs signed rank test (Two-tailed) W=10	*P*=0.125
	4AP (n=4)	0.4447		
Supplementary Fig.9b	Shock training			
	YFP (n=14)	0.1696	Two-way RM ANOVA $F_{(1, 29)} = 0.028$	*P*=0.8683
	ChR2 (n=17)	0.1969		
	Cue test			
	YFP (n=14)	0.0001	Mann Whitney test (Two-tailed) U=71.5	*P*=0.0599
	ChR2 (n=17)	0.001		
Supplementary Fig.9c	Context test			
	YFP (n=14)	0.1036	Mann Whitney test (Two-tailed) U=97.5	*P*=0.4046
	ChR2 (n=17)	0.004		
