# Supplementary material for: Gustatory thalamic neurons mediate aversive behaviors
Source: Nat Commun. 2025 Sep 26;16:8517. doi: 10.1038/s41467-025-63464-5 (PMC12475432; doi:10.1038/s41467-025-63464-5)
Supplement: Supplementary file 2 — Description of Additional Supplementary Files [file 41467_2025_63464_MOESM2_ESM.docx]

Description of Additional Supplementary File

Supplementary Movie 1. Open field test under 10 Hz light stimulation

Supplementary Movie 2. Open field test under 30 Hz light stimulation

Supplementary Movie 3. The projection pattern of Cck neurons in the VPMpc
