# Supplementary material for: Gustatory thalamic neurons mediate aversive behaviors
Source: Nat Commun. 2025 Sep 26;16:8517. doi: 10.1038/s41467-025-63464-5 (PMC12475432; doi:10.1038/s41467-025-63464-5)
Supplement: Supplementary file 6 — Reporting Summary [file 41467_2025_63464_MOESM6_ESM.pdf]

nature portfolio

Corresponding author(s): Richard D. Palmiter
Feng Cao

Last updated by author(s): Jul 30, 2025

# Reporting Summary

Nature Portfolio wishes to improve the reproducibility of the work that we publish. This form provides structure for consistency and transparency in reporting. For further information on Nature Portfolio policies, see our Editorial Policies and the Editorial Policy Checklist.

## Statistics

For all statistical analyses, confirm that the following items are present in the figure legend, table legend, main text, or Methods section.

| n/a | Confirmed | |
|---|---|---|
| [ ] | [x] | The exact sample size (*n*) for each experimental group/condition, given as a discrete number and unit of measurement |
| [ ] | [x] | A statement on whether measurements were taken from distinct samples or whether the same sample was measured repeatedly |
| [ ] | [x] | The statistical test(s) used AND whether they are one- or two-sided<br>*Only common tests should be described solely by name; describe more complex techniques in the Methods section.* |
| [ ] | [x] | A description of all covariates tested |
| [ ] | [x] | A description of any assumptions or corrections, such as tests of normality and adjustment for multiple comparisons |
| [ ] | [x] | A full description of the statistical parameters including central tendency (e.g. means) or other basic estimates (e.g. regression coefficient) AND variation (e.g. standard deviation) or associated estimates of uncertainty (e.g. confidence intervals) |
| [ ] | [x] | For null hypothesis testing, the test statistic (e.g. *F*, *t*, *r*) with confidence intervals, effect sizes, degrees of freedom and *P* value noted<br>*Give P values as exact values whenever suitable.* |
| [x] | [ ] | For Bayesian analysis, information on the choice of priors and Markov chain Monte Carlo settings |
| [x] | [ ] | For hierarchical and complex designs, identification of the appropriate level for tests and full reporting of outcomes |
| [x] | [ ] | Estimates of effect sizes (e.g. Cohen's *d*, Pearson's *r*), indicating how they were calculated |

*Our web collection on statistics for biologists contains articles on many of the points above.*

## Software and code

Policy information about availability of computer code

| | |
|---|---|
| Data collection | Data was collected with nVista 3.0 (Inscopix), pClamp 11 (Molecular Devices), MED-PC IV 4.2 (Med Associate), SR-LAB (San Diego Instruments), Ethovision XT15 (Noldus), Keyence BZ-710 microscope and Olympus FV-1200 confocal microscope. |
| Data analysis | Data was analyzed with IDPS 1.9.1 (Inscopix), Ethovision XT15 (Noldus), GraphPad Prism 10 (GraphPad Software), Fiji (NIH) and Matlab R2023b (MathWorks). |

For manuscripts utilizing custom algorithms or software that are central to the research but not yet described in published literature, software must be made available to editors and reviewers. We strongly encourage code deposition in a community repository (e.g. GitHub). See the Nature Portfolio guidelines for submitting code & software for further information.

## Data

Policy information about availability of data

All manuscripts must include a data availability statement. This statement should provide the following information, where applicable:

- Accession codes, unique identifiers, or web links for publicly available datasets
- A description of any restrictions on data availability
- For clinical datasets or third party data, please ensure that the statement adheres to our policy

Source data are provided with this manuscript. Other Data are available from the corresponding author upon reasonable request. The code used in this study is available from Zenodo (https://zenodo.org/records/16609026).

## Research involving human participants, their data, or biological material

Policy information about studies with human participants or human data. See also policy information about sex, gender (identity/presentation), and sexual orientation and race, ethnicity and racism.

| | |
|---|---|
| Reporting on sex and gender | N/A |
| Reporting on race, ethnicity, or other socially relevant groupings | N/A |
| Population characteristics | N/A |
| Recruitment | N/A |
| Ethics oversight | N/A |

Note that full information on the approval of the study protocol must also be provided in the manuscript.

# Field-specific reporting

Please select the one below that is the best fit for your research. If you are not sure, read the appropriate sections before making your selection.

[x] Life sciences  [ ] Behavioural & social sciences  [ ] Ecological, evolutionary & environmental sciences

For a reference copy of the document with all sections, see nature.com/documents/nr-reporting-summary-flat.pdf

# Life sciences study design

All studies must disclose on these points even when the disclosure is negative.

| | |
|---|---|
| Sample size | A power and sample size calculator was performed for the effective sample size using http://powerandsamplesize.com. |
| Data exclusions | Animals were excluded from analysis if the viral expression was inadequate, or off-target based on histology and imaging verification. |
| Replication | All experiments were performed with multiple animals and at least two cohorts of mice. Data from different cohorts were combined and analyzed together to ensure reproducibility. All n numbers were indicated in the figure legends. To ensure reproducibility, we included detailed protocol in the methods section. We also provided the sources of virus, antibodies and reagents in the manuscript. |
| Randomization | Animals from the same litter were randomly assigned to the control and the experimental groups with both males and females. |
| Blinding | Experimenters were blind to the group assignments and the treatments when conducted both experiments and analysis. |

# Reporting for specific materials, systems and methods

We require information from authors about some types of materials, experimental systems and methods used in many studies. Here, indicate whether each material, system or method listed is relevant to your study. If you are not sure if a list item applies to your research, read the appropriate section before selecting a response.

### Materials & experimental systems

| n/a | Involved in the study | |
|---|---|---|
| [ ] | [x] | Antibodies |
| [x] | [ ] | Eukaryotic cell lines |
| [x] | [ ] | Palaeontology and archaeology |
| [ ] | [x] | Animals and other organisms |
| [x] | [ ] | Clinical data |
| [x] | [ ] | Dual use research of concern |
| [x] | [ ] | Plants |

### Methods

| n/a | Involved in the study | |
|---|---|---|
| [x] | [ ] | ChIP-seq |
| [x] | [ ] | Flow cytometry |
| [x] | [ ] | MRI-based neuroimaging |

## Antibodies

| | |
|---|---|
| Antibodies used | Primary antibodies: chicken-anti-GFP (1:10000, Abcam, ab 13970), rabbit-anti-dsRed (1:2000, Takara, 632496).<br>Secondary antibodies: Alexa Fluor 488 donkey anti-chicken (1:500, Jackson ImmunoResearch, #703-545-155, 2340375), Alexa Fluor 594 donkey anti-rabbit (1:500, Jackson ImmunoResearch, #711-585-152, 2340621). |
| Validation | Antibodies used in this study were validated by the manufacturer and by previously published papers from the laboratory. |

By the manufacturer:
1. Chicken-anti-GFP (Abcam, ab13970): https://www.abcam.com/en-us/products/primary-antibodies/gfp-antibody-ab13970
2. Rabbit-anti-DsRed (Takara, 632496): https://www.takarabio.com/products/antibodies-and-elisa/fluorescent-protein-antibodies/red-fluorescent-protein-antibodies?catalog=632496
3. Alexa Fluor 488 donkey anti-chicken (Jackson ImmunoResearch, #2340375): hhttps://www.jacksonimmuno.com/catalog/products/703-545-155
4. Alexa Fluor 594 donkey anti-rabbit (Jackson ImmunoResearch, #2340621): https://www.jacksonimmuno.com/catalog/products/711-585-152

By previously published papers:
Chen, J. Y., Campos, C. A., Jarvie, B. C. & Palmiter, R. D. Parabrachial CGRP Neurons Establish and Sustain Aversive Taste Memories. Neuron 100, 891-899 e895 (2018). https://doi.org/10.1016/j.neuron.2018.09.032
Pauli, J. L. et al. Molecular and anatomical characterization of parabrachial neurons and their axonal projections. Elife 11, 81868 (2022). https://doi.org/10.7554/eLife.81868
Park, S., Zhu, A., Cao, F. & Palmiter, R. D. Parabrachial Calca neurons mediate second-order conditioning. Nat Commun 15, 9721 (2024). https://doi.org/10.1038/s41467-024-53977-w

## Animals and other research organisms

Policy information about studies involving animals; ARRIVE guidelines recommended for reporting animal research, and Sex and Gender in Research

| | |
|---|---|
| Laboratory animals | All animals (homozygous CckCre/Cre, Oprm1Cre/Cre and CalcaFLPo/FLPo mice) used in this study were backcrossed onto a C57BL/6J background for > 6 generations. Mice were maintained on a 12-h light/dark cycle (7 am-7 pm) with food and water ad libitum in a temperature- and humidity-controlled animal facility. |
| Wild animals | No wild animals were used in this study. |
| Reporting on sex | Both male and female mice were used in this study. No sex differences were noted. |
| Field-collected samples | N/A |
| Ethics oversight | All animal experimental protocols were approved by the Institutional Animals Care and Use Committee at the University of Washington (Protocol #2183–02). |

Note that full information on the approval of the study protocol must also be provided in the manuscript.

## Plants

| | |
|---|---|
| Seed stocks | N/A |
| Novel plant genotypes | N/A |
| Authentication | N/A |
